# Leadership practices and maternal and child health outcomes in Sub-Saharan Africa: a systematic review

**DOI:** 10.1186/s12913-026-14953-w

**Published:** 2026-06-27

**Authors:** Samuel Yao Mayeden, Ildiko Hoffmann, Myo Chit, Jussi P. Posti, Michael Lowery Wilson, Valerie R. Louis, Michael Marx, John Koku Awoonor-Wiliams, Alfred Edwin Yawson, Olaf Horstick, Andreas Deckert, Peter Dambach

**Affiliations:** 1 Heidelberg Institute of Global Health (HIGH), Faculty of Medicine and University Hospital, Heidelberg University, Heidelberg, Germany; 2 Injury Epidemiology and Prevention Research (IEP) Group, Department of Neuroscience, University of Turku, Turku, Finland; 3 Neurocenter, Department of Neurosurgery, Turku University Hospital and University of Turku, Turku, Finland; 4 Section for Oral Health, Heidelberg Institute of Global Health (HIGH), Faculty of Medicine and University Hospital, Heidelberg University, Heidelberg, Germany; 5 Ministry of Health, Accra, Ghana; 6 College of Health Sciences, University of Ghana, Accra, Ghana

**Keywords:** Leadership, Management, Primary health care, Maternal, Newborn and child health, Sub-Saharan Africa, Health systems strengthening, Quality of care

## Abstract

**Background:**

Maternal, newborn, and child health (MNCH) remain a major public health challenge in Sub-Saharan Africa (SSA), where preventable mortality persists despite global commitments to reduce deaths. Weak leadership and management within primary health care (PHC) systems contribute to inefficiencies, inequities in access, and suboptimal quality and continuity of care.

**Methods:**

This systematic review examined how leadership and management practices influence MNCH outcomes in SSA. The systematic review followed PRISMA 2020 guidelines and was conceptually informed by the WHO Health System Building Blocks framework and governance-oriented leadership concepts. Searches were conducted in PubMed/MEDLINE, Web of Science, Scopus, CINAHL, Embase, Google Scholar, and the WHO African Index Medicus (AIM) for peer-reviewed studies published between 1978 and July 2025. The protocol was registered in PROSPERO (CRD42024514304). Clinical trial number: Not applicable. Studies were eligible if they examined leadership or management practices in PHC settings and reported MNCH outcomes.

**Results:**

Thirteen studies from seven countries and one multi-country analysis met the inclusion criteria. Leadership approaches clustered around three domains: leadership capacity building and supportive supervision, participatory and accountable governance, and community engagement mechanisms. These approaches were consistently associated with measurable improvements in service coverage and quality. Reported gains included increases in antenatal care utilization (+ 12% to + 48%), skilled birth attendance (+ 10% to + 34%), and immunization completion rate (+ 8% to + 27%). Management practices including mentorship, routine monitoring, and data-driven decision-making strengthened provider performance, coordination, and service delivery efficiency.

**Conclusion:**

Leadership and management are critical system-level drivers of PHC performance and MNCH outcomes in SSA. Strengthening leadership and management capacity within PHC systems represents a high-impact strategy for improving service delivery and accelerating progress toward MNCH targets in SSA.

**Supplementary Information:**

The online version contains supplementary material available at 10.1186/s12913-026-14953-w.

## Evidence in context

Leadership and management are increasingly recognized as important determinants of maternal, newborn, and child health (MNCH) outcomes in Sub-Saharan Africa (SSA), yet they remain comparatively underexplored and underprioritized. Despite decades of investment in clinical training, vertical programs, and disease-specific interventions, persistent system challenges including weak governance, limited accountability, fragmented coordination, and workforce shortages, continue to undermine progress and widen inequities in access, quality, and outcomes. Evidence from global health research suggests that effective leadership within primary health care (PHC) systems strengthens accountability, coordination, provider competence, data-driven decision-making, and frontline health worker motivation for improving service coverage and care quality. However, the specific ways leadership and management practices influence MNCH outcomes in SSA remain insufficiently synthesized, limiting their visibility in policy design, implementation guidance, and resource allocation decisions. This systematic review addresses this gap by synthesizing evidence from studies conducted across seven SSA countries and one multi-country analysis to understand how district- and facility-level leadership and management approaches shape MNCH outcomes within PHC settings. By positioning leadership not merely as an administrative function but as a system-strengthening capacity, this systematic review provides contextually grounded evidence to inform policy design, guide strategic investments, and accelerate progress toward Sustainable Development Goal 3 (SDG 3): “Ensure healthy lives and promote well-being for all at all ages.” These findings highlight leadership and management as critical system-level levers within PHC that can translate policy intent into measurable MNCH gains when supported by adequate governance structures, resources, and community engagement mechanisms.

**Supplementary Information:**

The online version contains supplementary material available at 10.1186/s12913-026-14953-w.

## Background

Maternal, newborn, and child health (MNCH) remain among the most significant public health challenges in Sub-Saharan Africa (SSA). The region continues to record some of the highest maternal and newborn mortality rates globally [1, 2].

According to the most recent global estimates, the African region’s maternal mortality ratio declined from 727 per 100,000 live births in 2000 to 442 per 100,000 live births in 2023. Despite this progress, SSA alone accounted for approximately 70% of global maternal deaths in 2023 equivalent to around 182,000 maternal deaths [1–3]. Neonatal mortality continues to pose a considerable burden: African countries remain among those with the highest newborn death rates globally, with minimal annual reduction compared to other world regions [2].

Many of these maternal and newborn deaths are preventable, as most are associated with delays in seeking care, delays in reaching care or delay in receiving quality of care which is commonly referred to as the “Three Delays Model” [3, 4]. These patterns underscore persistent inequities in access to essential maternal and newborn services across the region. Women and newborns are disproportionately affected by systemic weaknesses, such as poor service readiness, fragmented referral systems, and insufficient continuity of care, contributing to elevated morbidity and mortality [5, 6]. These persistent barriers are shaped not only by clinical and infrastructural limitations, but also by weaknesses in health system governance, coordination, supervision and accountability. Leadership and management practices within PHC systems influence how resources are allocated, how frontline providers are supported, and how referral and service delivery systems function. In this context, leadership and management operate as system-level mechanisms that can strengthen responsiveness, continuity of care, and implementation of MNCH interventions within resource-constrained settings.

Over the past two decades, global initiatives such as Every Woman Every Child, the Global Strategy for Women’s Children’s and Adolescent’s Health, and the Sustainable Development Goals (SDGs) have renewed focus on reducing preventable maternal and child deaths. Particularly, SDG Target 3.1 calls for a global maternal mortality ratio below 70 per 100,000 live births, while Target 3.2 aims to eliminate preventable newborn and child deaths by 2030 [7, 8].

Despite these commitments, many SSA countries continue to face critical and persistent health-system bottlenecks, including shortages of skilled health workers, inadequate infrastructure and equipment, poor supply chains, and limited-service delivery especially at primary health care (PHC) level [8, 9]. Such constraints affect not only resource availability, but also how services are organized, coordinated and governed, limiting gains in MNCH outcomes.

Strong leadership and effective management within PHC are essential to building resilient health systems. Leadership practices that strengthen accountability, supportive supervision, workforce coordination and community responsiveness within PHC systems may contribute to improved MNCH service delivery and utilization [10]. Strengthening leadership capacity at the PHC level thus remains a critical strategic investment for accelerating progress toward MNCH and global health targets [11, 12].

## Methods

### Design and registration

To examine how leadership and management practices influence MNCH outcomes in SSA, we conducted a systematic review of published evidence. This approach enabled the integration of findings across diverse contexts, identification of recurring patterns, and an improved understanding of how leadership and management practices contribute to strengthening PHC systems. Our synthesis was conceptually informed by WHO Health System Building Blocks framework and governance-oriented leadership concepts, with attention to how leadership and management practices influence MNCH outcomes through accountability, supervision, coordination, workforce performance, and community engagement mechanisms. The systematic review followed the PRISMA 2020 guidelines [13] to ensure methodological transparency and rigor. The review protocol was registered with PROSPERO (CRD42024514304) on 17 April 2024 [14]. Clinical trial number: Not applicable.

### Search strategy

We conducted a comprehensive search for peer-reviewed studies published between September 1978 and July 2025. The start year corresponds to the Alma-Ata Declaration, which established primary health care as the foundation for achieving “health for all” [15] Extending the search through July 2025 allowed assessment of long-term trends and the evolution of leadership and management practices in PHC systems over nearly five decades.

The initial electronic search was conducted between 1 May and 31 July 2024. To ensure inclusion of the most recent evidence, the search was updated in July 2025 prior to final synthesis. Searches were conducted in PubMed/MEDLINE, Web of Science, Google Scholar, CINAHL, Scopus, Embase, and the WHO African Index Medicus (AIM). These databases were selected to provide broad disciplinary and geographic coverage across public health, health management, and social sciences.

Search terms were informed by the WHO Health System Building Blocks Framework [9] to ensure comprehensive coverage of leadership and management dimensions within PHC. For the purpose of this systematic review, leadership and management practices were defined as structured governance and organizational actions intended to influence planning, coordination, accountability, workforce performance, resource stewardship, or community engagement within PHC systems. This definition was informed by established health systems governance and leadership frameworks emphasizing accountability, decentralization, and performance management as core system functions [9, 16].

The complete PubMed/MEDLINE Boolean search strategy was:

(Leadership OR Governance OR Management OR Supervision OR “Supportive Supervision” OR Mentorship OR Coaching OR Guidance OR Training OR Teaching OR “Refresher Training” OR Coordination OR Accountability OR “Healthcare Management” OR “Health Service Administration”) AND (MNCH OR “Maternal Health” OR “Child Health” OR “Newborn Health” OR “Neonatal Health” OR “Maternal Child Health Services“[Mesh]) AND (“Community-Based Health” OR “Primary Health Care“[Mesh]) AND (“Sub-Saharan Africa” OR SSA OR Angola OR Benin OR Botswana OR Burkina Faso OR Burundi OR Cameroon OR Cape Verde OR Central African Republic OR Chad OR Comoros OR Congo OR Côte d’Ivoire OR Djibouti OR Equatorial Guinea OR Eritrea OR Ethiopia OR Gabon OR Gambia OR Ghana OR Guinea OR Guinea-Bissau OR Kenya OR Lesotho OR Liberia OR Madagascar OR Malawi OR Mali OR Mauritania OR Mauritius OR Mozambique OR Namibia OR Niger OR Nigeria OR Rwanda OR “Sao Tome and Principe“[Mesh] OR Senegal OR Somalia OR South Africa OR Sudan OR Swaziland OR Tanzania OR Togo OR Uganda OR Zambia OR Zimbabwe).

Because leadership and management practices are inconsistently labelled across PHC and health system literature, broader terms related to supervision, mentorship, training, coordination, accountability and health service administration were included to maximize our search sensitivity.

To capture MNCH-relevant studies, additional terms such as “Maternal Health,” “Newborn Health,” “Child Health,” “Maternal and Child Health Services,” and “Primary Health Care” [MeSH] were incorporated. Boolean operators (AND, OR) were used to combine concepts. Searches were limited to English-language studies conducted in SSA, as defined by the United Nations regional classification [7]. The language restriction was applied to ensure accuracy in data extraction and interpretation of complex leadership and governance constructs; however, no geographic restrictions were applied beyond SSA eligibility. Adapted search strategies for all other databases are provided in Supplementary File 1 in accordance with PRISMA 2020 reporting standards.

### Eligibility criteria

Studies were eligible if they examined how leadership, governance, or management practices influenced MNCH outcomes within PHC or community health systems.

We included quantitative, qualitative, and mixed-methods studies including randomized controlled trials, quasi-experimental studies, cohort studies, cross-sectional surveys, and case studies. Given the complex and system-level nature of leadership and management interventions in PHC, diverse study designs were included to capture both causal effects and contextual mechanisms. Randomized and quasi-experimental studies provided stronger causal inference regarding structured leadership and performance-based interventions, whereas cross-sectional and mixed-methods studies contributed insight into implementation processes, governance dynamics, and contextual influences.

Eligible studies were required to report measurable effects or clearly described associations between leadership and management practices and MNCH outcomes, such as antenatal care utilization, skilled birth attendance, institutional delivery, postnatal care coverage, immunization uptake, care-seeking for pregnancy complications, referral completion, or reductions in maternal and neonatal mortality. These outcomes were selected because they were the most consistently reported indicators across the included studies and reflect key domains of PHC performance, service utilization, continuity of care, and maternal and child health system responsiveness. Related outcomes such as institutional delivery, care-seeking for pregnancy complications, referral completion, and postnatal follow-up were considered eligible where they were reported in relation to leadership or management practices.

Only peer-reviewed, full-text journal articles published in English were included.

Studies were excluded if they focused solely on clinical services without leadership, governance or management components, or if clinical service delivery activities were not explicitly linked to decision-making authority, supervision structures, performance monitoring, governance, or management components. Commentaries, editorials, conference abstracts, policy briefs, and unpublished theses were also excluded.

### Screening and selection process

All retrieved citations were imported into Covidence [17], The platform automatically removed 353 duplicate records, with an additional six duplicates removed manually, resulting in 3,084 unique records for screening. Two reviewers (SYM and IH) independently screened titles and abstracts, excluding 3,036 records that did not meet inclusion criteria. Forty-eight full-text articles were assessed for eligibility, of which 13 met all inclusion criteria. Discrepancies were resolved through discussion and consensus.

### Quality appraisal

The methodological quality of all included studies was assessed using the Strengthening the Reporting of Observational Studies in Epidemiology (STROBE) checklist [18]. This tool evaluates clarity, transparency, and completeness of reporting across domains such as study design, data collection, results, and discussion. Each study was scored based on fulfillment of the checklist items, and results were expressed as percentage scores to indicate overall quality. Studies were categorized as high (> 85%), moderate (70–85%), or low (< 70%) quality. No studies were excluded based on quality assessment outcomes. However, variation in reporting completeness and leadership construct definitions limited direct comparison of effect magnitude across studies.

### Data extraction and synthesis

Data were extracted into a structured matrix capturing author, year, country, design, sample size, intervention type, leadership domain, and key MNCH outcomes. Two reviewers (SYM and IH) independently extracted data and discrepancies were resolved through consensus. Findings were synthesized using narrative and thematic approaches, with attention to leadership mechanisms (e.g., community engagement, capacity building, accountability) and their observed impacts on MNCH indicators. Due to heterogeneity in study designs and outcome measures, meta-analysis was not performed. Studies were grouped by intervention type and MNCH outcome domain to enable structured comparison across contexts, and variation in evidence strength was considered according to study design. Where possible, extracted leadership practices were mapped to intermediate system mechanisms, including provider competence, accountability, coordination, community trust, and service continuity, to support a system-oriented interpretation of our findings.

## Results

### Search results and characteristics

**Fig. 1:**
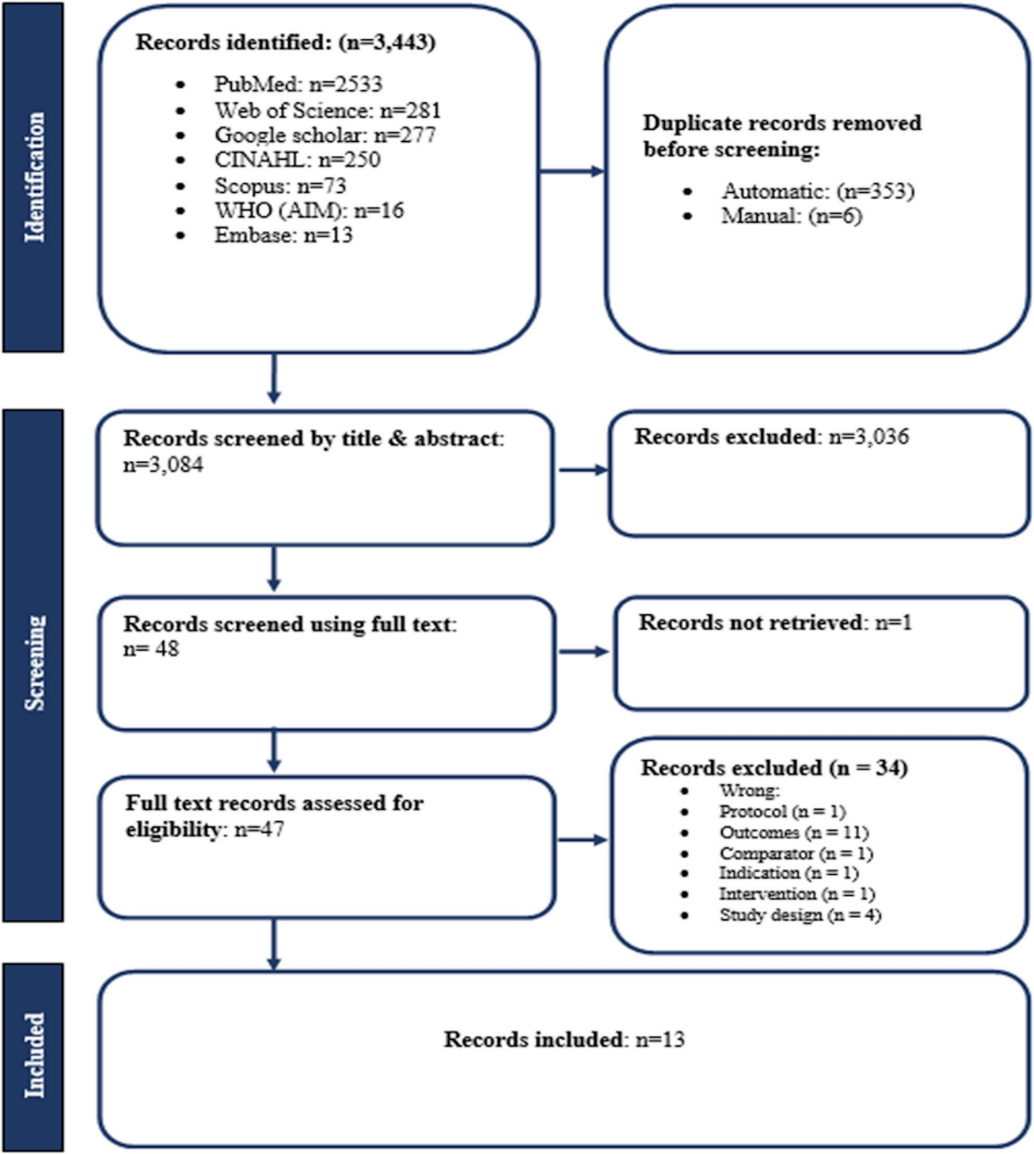
PRISMA flow diagram illustrating the identification, screening, and inclusion of studies in the systematic review

A total of 3,443 records were identified through the search. After removing duplicates, 3,084 studies were screened. Thirteen studies met the inclusion criteria for final synthesis. The study screening and selection process is presented in Fig. 1. These studies represented seven SSA countries and one multi-country analysis. The characteristics summarized in Table 1 reinforce that existing evidence is still limited and unevenly distributed across the region, with most studies coming from East Africa and using cross-sectional or mixed methods designs.

### Geographic distribution of included studies

**Fig. 2:**
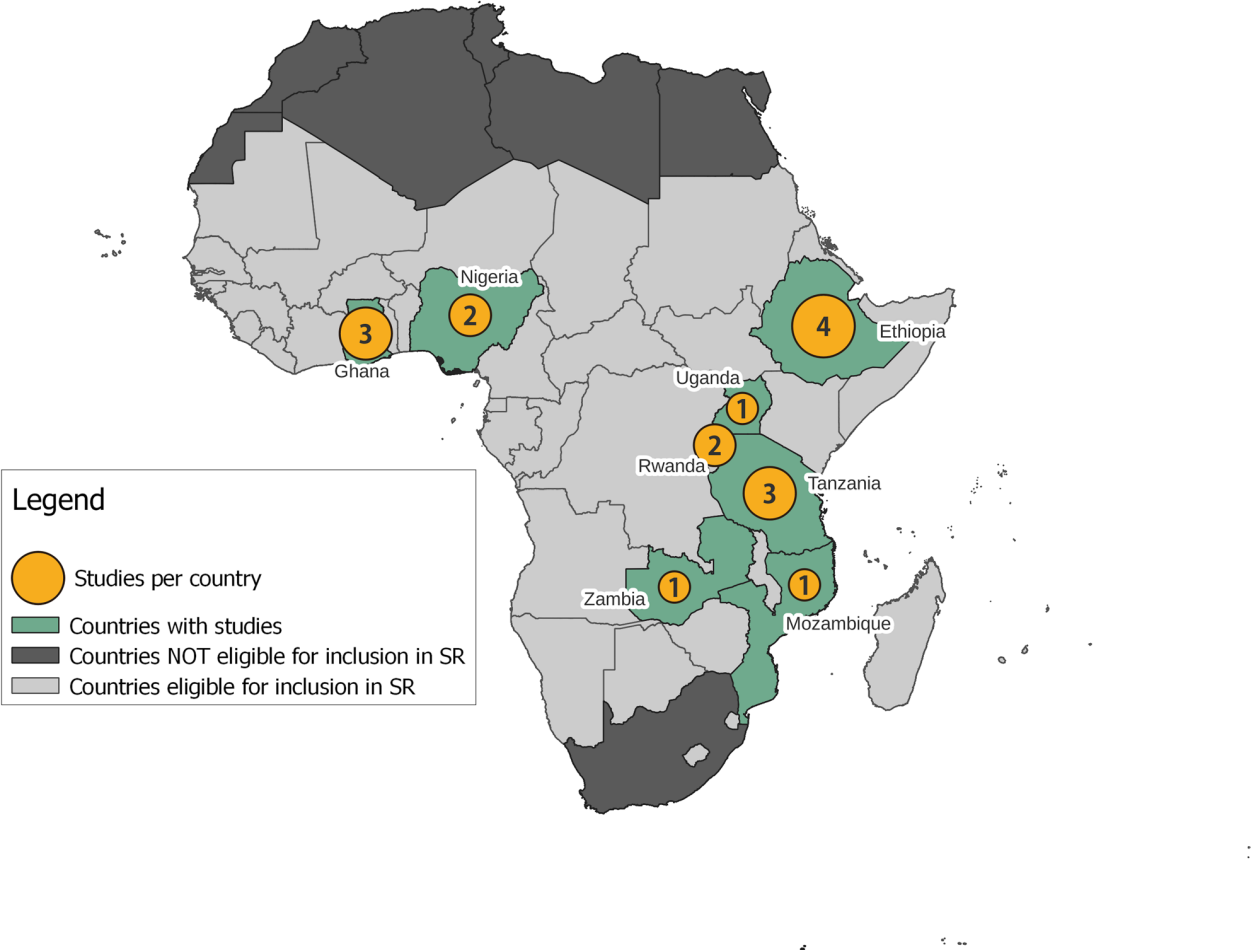
Geographic distribution of included studies on leadership and management practices affecting MNCH outcomes in Sub-Saharan Africa

Geographic analysis showed that Ethiopia (*n* = 4), Tanzania (*n* = 3), and Ghana (*n* = 3) contributed the largest number of studies, followed by Nigeria (*n* = 2), Rwanda (*n* = 1), and Uganda (*n* = 1). One multi-country study was also included [12]. Variations in publication patterns were observed, with Ethiopia, Tanzania, and Ghana contributing the highest concentration of studies. The geographical distribution of included study locations is presented in Fig. 2.

### Study design and methodological characteristics

Study designs varied widely, including cross-sectional, quasi-experimental, mixed-methods, and randomized controlled trials, indicating methodological diversity. The study designs, sample sizes, and methodological characteristics of the included studies are summarized in Table 1.

### Study quality appraisal

The reporting quality of the observational studies included was assessed using the Strengthening the Reporting of Observational Studies in Epidemiology (STROBE) checklist [18]. The checklist was used to evaluate the presence of key reporting elements such as study design, sampling, data collection, analysis, and interpretation. The tool does not assess methodological rigor or risk of bias but provides an indication of completeness and transparency in reporting across studies.

Studies included demonstrated moderate to high reporting quality. Most clearly articulated research objectives, applied appropriate designs, and provided transparent descriptions of data collection and analysis procedures. High-quality studies such as [12, 19, 20] exhibited robust methodological frameworks and comprehensive reporting.

**Fig. 3:**
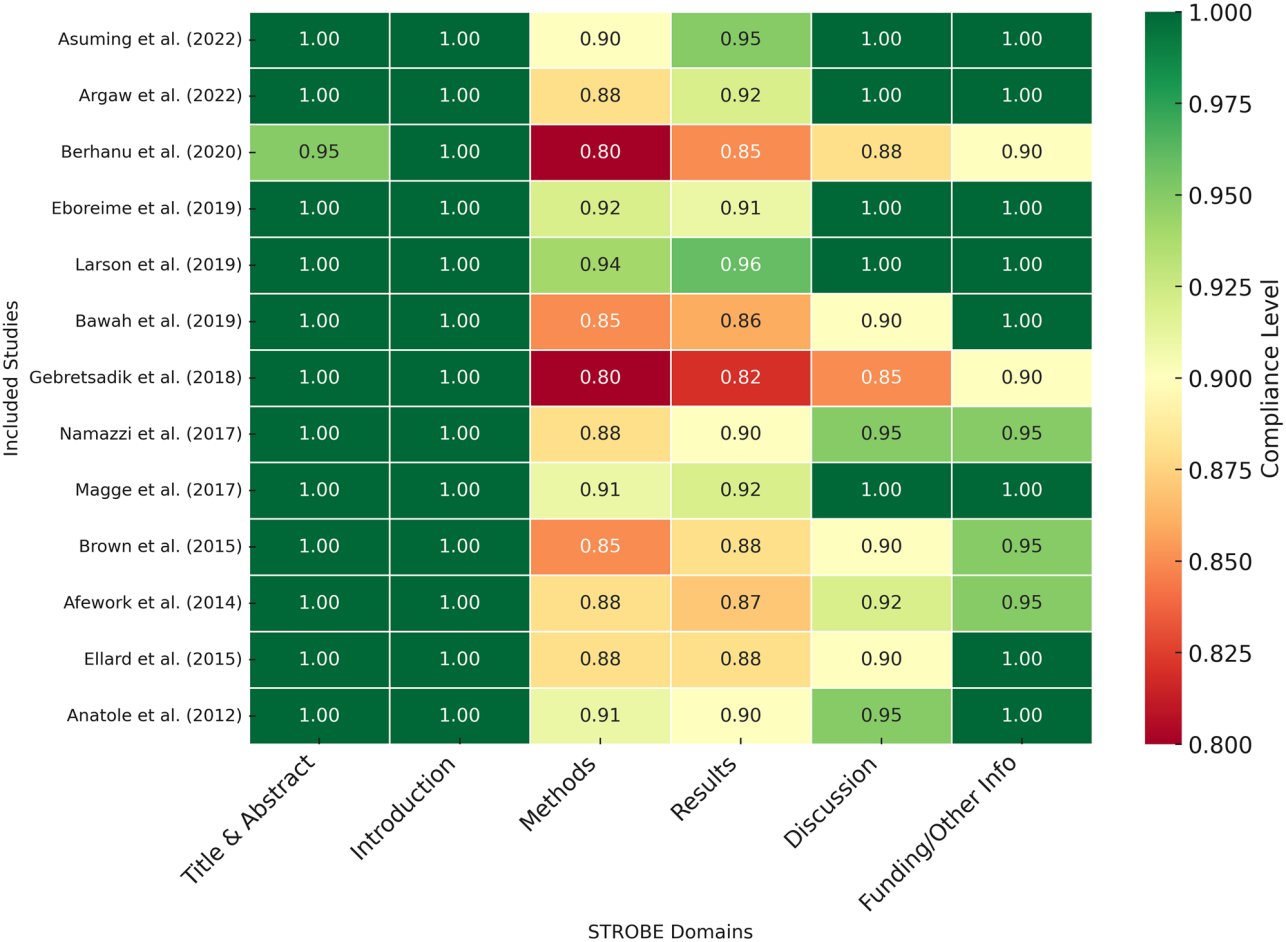
Heatmap of methodological quality based on the STROBE checklist across six reporting domains: Title and Abstract, Introduction, Methods, Results, Discussion, and Funding. Green indicates full compliance, yellow indicates partial compliance, and red indicates lower reporting quality

Minor reporting weaknesses were observed in a few studies, particularly in relation to sampling descriptions and statistical procedures. However, these shortcomings did not compromise the relevance or interpretability of the findings. No studies were excluded based on reporting quality, supporting the credibility and representativeness of the synthesized evidence. Figure 3 presents the overall reporting quality of included studies using the STROBE checklist. Most studies demonstrated moderate to high reporting completeness across the six domains.

**Table 1:** Characteristics of eligible articles

ID	Author & Year	Duration	Country	Study Setting	Study Population	Study Design	Sample Size
1	Anatole et al. (2012) [21]	2010	Rwanda	Southern Kayonza & Kirehe	Nurses incl. Nurse-Mentors & Health Center Staff	Mixed methods	21 Health Centers, 15 Nurses
2	Ellard et al. (2015) [22]	2011–2014	Tanzania	Rural	AMOs, COs, and NMWs	Pre-examination/post survey/mixed	36 PHFs
3	LARSON et al. (2019) [19]	2012–2016	Tanzania	Districts in Pwani Region	HCP & Patients	Cluster-randomized controlled	Facilities: 24 primary care clinics, Providers: 70 at baseline; 119 at endline, Patients: 784 at baseline; 886 at endline
4	Asuming et al. (2022) [20]	2010–2015	Ghana	Upper East Region	Children < 0–5 years	Two-stage random sample survey	Baseline: 2,697 children from 2,420 households Endline: 3,922 children from 2,719 households
5	Namazzi et al. (2017) [23]	2012–2015	Uganda	Kamuli, Pallisa, & Kibuku	CHWs	Quasi-experimental	Post: 402 CHWs, Mini survey: 513 CHWs, Baseline and final survey: 2,293 and 1,946 mothers
6	Magge et al. (2017) [12]	2011–2015	Multi Country	Rural	Newborns	Mixed methods	2,183 households (Baseline) 2,178 households (Endline)
7	Afework et al. (2014) [24]	2012	Ethiopia	Wukro & Butajira	Women 15–49 years & delivered within the last two years	Community-based cross-sectional survey	4,949 women
8	Eboreime et al. (2019) [25]	2016 & 2017	Nigeria	Chikun LGA; Kaduna State	Local Government Actors: health managers, donors, civil society representatives, and healthcare providers	Cross-sectional survey	75 PHC facilities 2 Secondary HFs
9	Argaw et al. (2022) [26]	2017–2018	Ethiopia	Amhara, Oromia, Tigray, and SNNP	HCP who focused on MNCH services	Cross-sectional survey	454 participants
10	Gebretsadik et al. (2018) [27]	2017	Ethiopia	Sidama Zone: Shebadino, Boricha, Wonsho, Dale, & Loka Abaya	Mothers who had a live birth in the last 6 months	Community-based cross-sectional survey	2,040
11	Bawah et al. (2019) [16]	2010–2015	Ghana	Builsa, Bongo, & Garu-Tempane: Upper East Region	Women aged 15–49	Quasi-experimental	Baseline = 5,511 women & 7,410 children Endline = 5,914 Women, & 7,044 children
12	Brown et al. (2015) [28]	2012–2013	Nigeria	Ibadan	Children < 0–12 months	Randomized controlled trial	605 children
13	Berhanu et al. (2020) [29]	2016–2019	Ethiopia	Amhara, SNNP, Oromia, & Tigray	Children > 5 years	Quantitative/Cross sectional	Baseline 5,773 households with 2,874 U5, Endline 10,788 households with 5,639 U5

PHC = Primary Health Care; HCP = Health Care Provider; CHW = Community Health Worker; RCTT = Randomized Controlled Trial

### Positive leadership and management practices

Positive leadership and management practices were consistently associated with improvements in MNCH service delivery and utilization across PHC systems. Across the included studies, these practices clustered into three leadership domains: (1) leadership capacity building and supportive supervision, (2) participatory and accountable governance, and (3) community engagement mechanisms [9, 12]. These were interpreted within a systems-oriented framework in which leadership practices influence MNCH outcomes through intermediate mechanisms such as provider competence, accountability, coordination and community trust. Community engagement mechanisms emerged as a key success factor. In Ghana, community participation paired with strengthened emergency referral systems resulted in reduced childhood illnesses and improved utilization of maternal services [20]. Similarly, district health teams in Uganda enhanced community health worker (CHW) performance by providing structured supervision and resources that led to increased institutional deliveries and improved maternal health outcomes [23].

Leadership capacity building and accountability mechanisms also strengthened leadership effectiveness. In Tanzania, in-service training and mentorship strengthened provider competence and newborn counseling while routine monitoring and data-driven decision-making increased antenatal care attendance and facility-based deliveries across multiple countries [19]. These findings underscore the importance of continuous learning and accountability as drivers of service quality [12].

Participatory and accountable governance further enhanced PHC system performance. In Nigeria, participatory action research (PAR) created shared ownership between government officials, civil society, and frontline providers, improving decision-making and fostering alignment between interventions and community needs [25]. This inclusive leadership model strengthened service delivery and responsiveness to local priorities.

Collectively, these findings demonstrate that leadership approaches grounded in trust, transparency, collaboration and shared accountability contribute to measurable improvements in MNCH outcomes. The relationship between leadership approaches and MNCH outcomes, including improvements in antenatal care attendance, skilled delivery, and immunization uptake, is summarized in Table 2.

### Barriers and weaknesses in leadership and management

Despite encouraging progress, several systemic barriers limit leadership effectiveness. Infrastructure and resource challenges were the most frequently cited obstacles. Studies noted that inadequate staffing, poor facility infrastructure, and weak supply chains hindered timely access to essential MNCH services even where leadership interventions were introduced [22]. In Tanzania, for example, maternal and neonatal outcomes remained constrained when limited referral and facility readiness slowed emergency response within integrated PHC systems.

Workforce misalignment further undermined efficiency. Poor task allocation, overlapping staff roles, and inconsistent supervisory structures negatively affected quality of care, particularly for postnatal and neonatal services [27]. These findings highlight the need for clear role definition, cohesive team structures, and strengthened performance management systems.

Weak community engagement reduced service uptake. In Ethiopia, limited involvement of communities in PHC governance was associated with lower use of essential services including immunization and postnatal care [29]. These findings suggest that leadership models are likely to improve MNCH outcomes when community participation is combined with supportive supervision, accountability mechanisms and adequate system-level resources.

### Outcomes on MNCH Indicators

Leadership and management practices demonstrated a clear and measurable association with MNCH service coverage, utilization, and continuity indicators across the reviewed studies. For maternal health outcomes, leadership-led interventions such as home visits by health extension workers [24] and decentralized decision-making at health centers [26] were more associated with significant increases in antenatal care utilization and skilled birth attendance. Across studies, improvements ranged from + 12% to + 48% for ANC utilization and + 10% to + 34% for skilled delivery. Institutional delivery, postnatal follow-up, care-seeking for pregnancy complications, referral completion, and immunization uptake were considered where reported, but antenatal care utilization, skilled birth attendance, immunization completion rate, and neonatal outcomes were the most consistently reported indicators across studies.

For child health outcomes, community-based governance and emergency referral systems in Ghana [20] markedly reduced the prevalence of common childhood illnesses, while the use of mobile health (mHealth) technologies in Nigeria [28] improved immunization completion rates and strengthened continuity of care.

For newborn health outcomes, performance-based and data-driven interventions implemented across multiple countries contributed to reductions in neonatal mortality and improvements in postnatal care coverage [12]. In Ghana, these approaches contributed to reductions in neonatal mortality and improvements in postnatal care coverage. These strategies enhanced service readiness, provider accountability, and follow-up coverage, demonstrating their role in improving continuity and equity of care [16].

Collectively, these findings indicate that effective leadership practices reinforce PHC systems, enhance accountability, and drive measurable improvements in MNCH outcomes.

### Conceptual linkages between leadership practices and MNCH outcomes

**Fig. 4:**
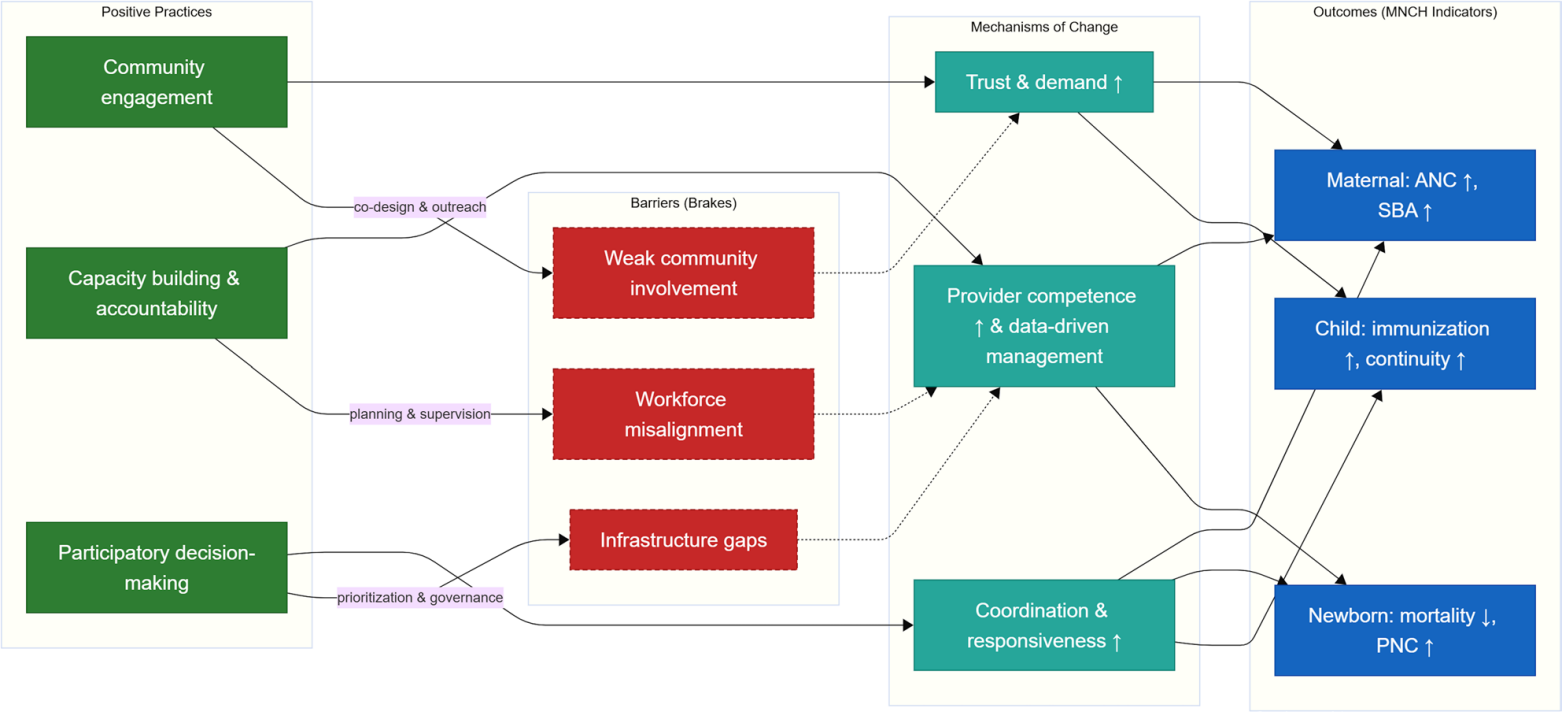
Conceptual framework illustrating pathways through which leadership and management practices influence MNCH outcomes in Sub-Saharan Africa. Positive leadership practices strengthen health system mechanisms, leading to improved outcomes, while systemic barriers act as constraints

The conceptual framework synthesizing relationships between leadership practices and MNCH outcomes is presented in Fig. 4 and reflects a systems-oriented theory of change informed by the WHO Health Systems Building Blocks framework and governance-oriented leadership concepts. Leadership practices such as participatory governance, supportive supervision, mentorship and data-driven management influence intermediate system mechanisms including provider competence, coordination, accountability, and community trust. These mechanisms, in turn, improve service coverage, quality, and continuity, ultimately contributing to improved MNCH outcomes. For instance, district supervision systems in Uganda strengthened community health worker performance and increased institutional deliveries, while community-participatory governance processes in Ghana improved referral coordination and maternal service utilization. Similarly, mentorship and performance monitoring initiatives in Tanzania and Ethiopia enhanced provider competence and antenatal care utilization. This conceptual theory-of-change pathway illustrates how leadership operates as a system-strengthening function within PHC rather than as a standalone intervention.

Conversely, systemic barriers such as infrastructure gaps, workforce misalignment, and weak community involvement act as constraints. This framework suggests that these barriers can be mitigated through leadership strategies that promote accountability, data-driven decision-making, and adaptive governance.

Feedback loops within this model illustrate continuous learning processes, reinforcing the importance of evidence-informed leadership for sustainable health system strengthening. 

**Table 2:** Findings on leadership and management factors and related impact on MNCH and recommended policy implication in SSA

ID	Author & Year	Location	Positive Factors	Negative Factors	Impact on MNCH Indicators	Policy Implication
**1**	Anatole et al. (2012) [21]	Rwanda	Adapted WHO antenatal care protocols, engagement with community health workers	None noted	Increase in ANC utilization, though average visits below WHO target; low birth weight issues	Calls for reforms to strengthen community-level health services and trust in health extension workers
**2**	Ellard et al. (2015) [22]	Tanzania	Collaboration with MOH to integrate clinicians in rural services, focus on comprehensive emergency maternal and neonatal care	Inadequate infrastructure and resources	Slight decrease in maternal mortality rates	Need for policy adjustments to improve rural health infrastructure and resource availability
**3**	Larson et al. (2019) [19]	Tanzania	In-service training, mentorship, and community involvement to enhance service quality	None noted	Improved newborn counseling, increased provider knowledge	Suggested improvements in healthcare infrastructure and provider training
**4**	Asuming et al. (2022) [20]	Ghana	Community engagement, governance enhancement, effective emergency referral systems	None noted	Significant reduction in fever and cough among children under five	Advocates for continued investment in leadership development and health infrastructure
**5**	Namazzi et al. (2017) [23]	Uganda	Support for community health workers by district health teams, structured community engagement	None noted	Increase in institutional deliveries	Proposes sustainable incentives for CHWs and better integration into formal health systems
**6**	Magge et al. (2017) [12]	Multi-country	Performance reviews, data-driven decision-making across multiple countries	Sustainability concerns due to dependency on external support	Increases in ANC visits and health facility deliveries	Emphasizes the need for ongoing support and capacity building for sustainable leadership
**7**	Afework et al. (2014)[24]	Ethiopia	Home visits by health extension workers, national program support for health access	None noted	Improved ANC utilization by threefold	Highlights the importance of facility readiness and birth preparedness to enhance maternal health
**8**	Eboreime et al. (2019) [25]	Nigeria	Training and evidence-based interventions, participatory action research (PAR) methodologies for collaborative decision-making	None noted	Improvements in maternal and child health care quality	Calls for continuous improvement cycles and institutionalizing participatory models
**9**	Argaw et al. (2022) [26]	Ethiopia	Training and decentralized decision-making at health centers, enhanced accountability	None noted	Increased skilled birth attendance and maternal-child health performance	Stresses strengthening local health center leadership for long-term health system resilience
**10**	Gebretsadik et al. (2018) [27]	Ethiopia	Home-based neonatal care, integrated monitoring and task-sharing at the community level	Delay in post-birth home visits, overlapping responsibilities of health workers	Positive impact on neonatal care practices, increase in timely postnatal visits	Urges better supervision of health programs and policy adjustments for effective neonatal care
**11**	Bawah et al. (2019) [16]	Ghana	Data-driven decision-making, comprehensive stakeholder engagement	None noted	Significant reduction in neonatal mortality, improvements in ANC and skilled birth attendance	Recommends scaling up comprehensive health systems strengthening strategies
**12**	Brown et al. (2015) [28]	Nigeria	Implementation of cellphone reminder/recall systems and primary health care immunization training	None noted	Substantial increase in immunization completion rates	Promotes the adoption of mobile health technologies in low-resource settings
**13**	Berhanu et al. (2020) [29]	Ethiopia	Ownership and integrated community engagement in child health services	Inadequate community engagement leading to decreased care utilization	No significant change in utilization of care for sick children under 5	Suggests more robust community engagement and sustained funding for child health services

## Discussion

This systematic review addresses an important evidence gap by bringing together research on leadership and management practices within PHC systems in SSA and examining their influence on MNCH outcomes. Three key themes emerged: (1) leadership capacity building and supportive supervision were consistently linked to improvements in service delivery and provider performance; (2) participatory and accountable governance strengthened decision-making, resource stewardship, and teamwork; and (3) community engagement enhanced trust, service utilization, and contextual relevance of MNCH interventions. These findings highlight that leadership at the PHC level is not merely administrative, but it plays a strategic role in improving health system performance and MNCH outcomes in the region.

Across countries, leadership practices operated through a systems-oriented theory-of-change pathway involving intermediate system mechanisms, including provider competence, accountability, coordination, and community trust, which explain how PHC leadership translates into MNCH gains. For instance, district health teams in Uganda strengthened CHW performance through ongoing supervision and mentorship, which was associated with increased institutional deliveries, while in Ghana community participation combined with strengthened referral systems improved maternal service utilization and reduced childhood illness. In Tanzania, mentorship and in-service training improved provider competence in newborn care.

A key finding of this review is the central role of district-level leadership in improving PHC system performance. Some studies demonstrate how giving districts more decision-making authority and using performance-based monitoring can improve key MNCH indicators, including antenatal care attendance and skilled birth delivery [12, 26]. These examples show that when local leaders are trusted to make decisions and are held accountable for results, services become more responsive to community needs. This finding aligns with WHO’s vision for primary healthcare leadership that promotes accountability, supports frontline health workers, and focuses on local priorities.

Evidence from different study designs contributed complementary insights into leadership effectiveness. Randomized and quasi-experimental studies provided stronger support for structured supervision, mentorship, and performance-monitoring approaches, whereas cross-sectional and mixed-methods studies primarily clarified implementation pathways and contextual drivers influencing leadership effectiveness [19].

District-level leadership also plays a critical role in connecting national health policies with local implementation. Structured supervision, mentorship, and participatory decision-making mechanisms helped ensure that health programs were adapted to local realities, improving coordination and service delivery [23]. Similarly participatory decision-making, where health leaders, providers, and community stakeholders jointly plan and solve service delivery challenges, improved coordination and reduced bottlenecks [25]. These findings confirm that empowered leadership fosters collaboration across sectors and makes PHC more responsive and accountable.

Our systematic review revealed persistent barriers that limit how effectively district leaders can manage PHC systems. Inadequate infrastructure and chronic resource shortages remain among the most significant challenges. As noted, many district managers lack decision-making authority or stable funding, making it difficult to sustain improvements or address service delivery gaps. Without decentralization of resources alongside leadership responsibility, local systems cannot maintain gains [22].

However, the effectiveness of leadership interventions also varied across settings depending on governance arrangements and resource availability. For instance, Ethiopia demonstrated improvements in antenatal care utilization where decentralization was combined with performance monitoring systems. In contrast, studies from Tanzania suggested that infrastructure gaps and resource limitations constrained the sustainability of leadership-driven improvements in maternal services. Evidence from Nigeria also indicated that participatory governance improved coordination and responsiveness but required strong local implementation capacity to sustain gains. These contrasts suggest that leadership interventions were more effective when managerial authority was matched by adequate resources, supervision capacity, and implementation support.

Another major issue is workforce misalignment. In many contexts, health workers face overlapping duties or unclear responsibilities, which reduces efficiency and can delay essential care. This reflects a broader leadership need for clearer staffing structures, supervision mechanisms, and role accountability [27]. For instance, limited community involvement was associated with lower uptake of immunization and maternal health services. Leadership that prioritizes community partnerships builds trust and encourages consistent use of available services [29]. These findings suggest that leadership influences MNCH outcomes not only through formal authority structures but also through relational mechanisms such as trust-building, reinforcement of accountability, and coordination across health system actors.

Our systematic review highlights the value of structured leadership development programs, including mentorship, in-service training, and performance review systems [19]. Evidence from Tanzania showed that mentorship improved newborn care competence, while performance monitoring in Ethiopia increased ANC utilization. Institutionalizing such programs could help sustain improvements by ensuring that PHC leaders have both skills and confidence to address emerging challenges [12]. Evidence from Ghana also demonstrated that community involvement in decision-making reduced child illnesses by overcoming local barriers to care. These examples show that strong leadership is not only about management structures, but also about relationships, motivation, and shared accountability [20]. Despite these consistent patterns, variability in reporting of leadership constructs and contextual factors limited systematic comparison of effect magnitude across intervention types and study designs.

Governance frameworks may need to evolve to better support district-level leadership. As observed across included studies, decentralized systems allow health managers to tailor interventions to local contexts. To strengthen such systems, national policies should focus on fair resource distribution, local capacity building, and clear lines of accountability [26]. To maximize this potential, national policies should prioritize resource equity, local capacity development, and formal accountability channels. Performance-based leadership approaches offer promising opportunities to link leadership activities to measurable improvements in MNCH outcomes, enabling leaders to identify bottlenecks and adjust strategies in real time [12]. Overall, the evidence positions leadership and management as system-level levers that translate policy intent into measurable MNCH gains when supported by adequate resources, governance capacity, and community engagement mechanisms.

This systematic review reinforces the message that leadership and management are not optional “soft skills” but foundational levers for health system performance. We recommend that governments, donors, and training institutions invest in competency-based leadership development tailored to PHC management. This should strengthen technical, managerial, and relational skills to help leaders address systemic barriers and build trust with communities. Policies should also ensure that leaders have both authority and resources to act effectively. Finally, sustained investments in infrastructure and health workforce planning are essential to support resilient PHC systems.

Future research should explore innovative leadership models, including digital governance and community-driven approaches, to improve accountability and coordination. By combining effective leadership, robust management, and community participation, PHC systems across SSA may be better positioned to deliver equitable and sustained improvements in MNCH outcomes.

## Strengths and limitations

This systematic review offers one of the few systematic examinations of leadership and management influences on maternal, newborn, and child health outcomes within primary healthcare systems in Sub-Saharan Africa. Strengths include a rigorous multi-database search, PROSPERO registration, adherence to PRISMA standards, and use of the STROBE checklist for quality appraisal. However, the evidence base remains limited, with few eligible studies and considerable variation in study design and reported outcomes, restricting comparability and preventing meta-analysis. A further limitation was the absence of standardized leadership and management metrics across studies, which limited direct comparison of leadership effectiveness and reduced the ability to assess effect magnitude across intervention types and settings. Limiting inclusion to English-language studies may have excluded relevant research. Despite these constraints, the review provides meaningful insights for strengthening leadership capacity to enhance MNCH outcomes in the region.

## Conclusions and recommendations

This systematic review demonstrates that leadership and management practices are important system-level drivers of PHC performance and MNCH outcomes in SSA. Across the included studies, leadership approaches clustered around three domains: leadership capacity building and supportive supervision, participatory and accountable governance, and community engagement mechanisms. These practices influence MNCH outcomes through intermediate system mechanisms such as improved provider competence, coordination, accountability, and community trust.

Evidence from diverse study designs suggests that district-level leadership, particularly when supported by structured supervision, mentorship, and performance management, can strengthen service delivery and increase utilization of essential MNCH services. However, the effectiveness and sustainability of these leadership approaches depend on contextual conditions, including governance arrangements, resource availability, and local implementation capacity.

Strengthening leadership capacity within PHC systems, alongside investments in workforce development, governance structures, and community engagement, may therefore represent a practical strategy for improving service delivery and accelerating progress toward MNCH targets and SDG 3 in SSA. Recognizing leadership and management as core system capabilities rather than administrative functions may offer a scalable and cost-effective pathway for strengthening PHC systems and advancing MNCH outcomes across the region and other resource-constrained settings. 

## Supplementary Information

Below is the link to the electronic supplementary material.

Supplementary Material 1: Search strategy for electronic databases

## Data Availability

All data supporting the findings of this study are included within the article and its supplementary files. No new primary data was generated for this systematic review.

## Abbreviations

ANC: Antenatal Care
AIM: African Index Medicus
BMC: BioMed Central
BMFTR: German Federal Ministry of Research, Technology and Space
CINAHL: Cumulative Index to Nursing and Allied Health Literature
CRD: Centre for Reviews and Dissemination
DASH: Research Network for Design and Evaluation of Adolescent Health Interventions and Policies in Sub-Saharan Africa
LMICs: Low-and Middle-Income Countries
mHealth: Mobile Health
MNCH: Maternal, Newborn, and Child Health
PHC: Primary Health Care
PNC: Postnatal Care
PRISMA: Preferred Reporting Items for Systematic Reviews and Meta-Analyses
PROSPERO: International Prospective Register of Systematic Reviews
RCT: Randomized Controlled Trial
RHISSA: Research Networks for Health Innovations in Sub-Saharan Africa
SBA: Skilled Birth Attendance
SDGs: Sustainable Development Goals
SSA: Sub-Saharan Africa
STROBE: Strengthening the Reporting of Observational Studies in Epidemiology
WHO: World Health Organization
