# Supplementary material for: Leadership practices and maternal and child health outcomes in Sub-Saharan Africa: a systematic review
Source: BMC Health Serv Res. 2026 Jun 27;26:878. doi: 10.1186/s12913-026-14953-w (PMC13312565; doi:10.1186/s12913-026-14953-w)
Supplement: Supplementary file 1 — Supplementary Material 1: Search strategy for electronic databases [file 12913_2026_14953_MOESM1_ESM.docx]

**SUPPLEMENTARY FILE 1. Search Strings Used Across Six Electronic Databases**

Our electronic search was initially conducted between May and July 2024 and updated in July 2025 prior to final synthesis.

| Database | Number of Results | Search String |
| --- | --- | --- |
| PubMed/MEDLINE | 2,533 | (Leadership OR Governance OR Supervision OR Coordination OR Healthcare Management) AND (MNCH OR Maternal Health OR Child Health OR Newborn Health OR Neonatal Health OR “Maternal Child Health Services”[MeSH]) AND (Community-based health OR “Primary Health Care”[MeSH]) AND (Sub-Saharan Africa OR SSA OR Angola OR Benin OR Botswana OR Burkina Faso OR Burundi OR Cameroon OR Cape Verde OR Central African Republic OR Chad OR Comoros OR Brazzaville OR Congo OR Côte d'Ivoire OR Djibouti OR Equatorial Guinea OR Eritrea OR Ethiopia OR Gabon OR Gambia OR Ghana OR Guinea OR Guinea Bissau OR Kenya OR Lesotho OR Liberia OR Madagascar OR Malawi OR Mali OR Mauritania OR Mauritius OR Mozambique OR Namibia OR Niger OR Nigeria OR Rwanda OR “Sao Tome and Principe”[MeSH] OR Senegal OR Somalia OR South Africa OR Sudan OR Swaziland OR Tanzania OR Togo OR Uganda OR Zambia OR Zimbabwe) |
| Google Scholar | 50,500 (first 277 screened) | (Leadership OR Governance OR Supervision OR Coordination OR Healthcare Management) AND (MNCH OR Maternal Health OR Child Health OR Newborn Health OR Neonatal Health OR “Maternal Child Health Services”) AND (Community-based health OR “Primary Health Care”) AND (Sub-Saharan Africa OR SSA OR Angola OR Benin OR Botswana OR Burkina Faso OR Burundi OR Cameroon OR Cape Verde OR Central African Republic OR Chad OR Comoros OR Brazzaville OR Congo OR Côte d'Ivoire OR Djibouti OR Equatorial Guinea OR Eritrea OR Ethiopia OR Gabon OR Gambia OR Ghana OR Guinea OR Guinea Bissau OR Kenya OR Lesotho OR Liberia OR Madagascar OR Malawi OR Mali OR Mauritania OR Mauritius OR Mozambique OR Namibia OR Niger OR Nigeria OR Rwanda OR “Sao Tome and Principe” OR Senegal OR Somalia OR South Africa OR Sudan OR Swaziland OR Tanzania OR Togo OR Uganda OR Zambia OR Zimbabwe) |
| Web of Science (Clarivate) | 281 | (Leadership OR Governance OR Supervision OR Coordination OR Healthcare Management) AND (MNCH OR Maternal Health OR Child Health OR Newborn Health OR Neonatal Health OR “Maternal Child Health Services”) AND (Community-based health OR “Primary Health Care”) AND (Sub-Saharan Africa OR SSA OR Angola OR Benin OR Botswana OR Burkina Faso OR Burundi OR Cameroon OR Cape Verde OR Central African Republic OR Chad OR Comoros OR Brazzaville OR Congo OR Côte d'Ivoire OR Djibouti OR Equatorial Guinea OR Eritrea OR Ethiopia OR Gabon OR Gambia OR Ghana OR Guinea OR Guinea Bissau OR Kenya OR Lesotho OR Liberia OR Madagascar OR Malawi OR Mali OR Mauritania OR Mauritius OR Mozambique OR Namibia OR Niger OR Nigeria OR Rwanda OR “Sao Tome and Principe” OR Senegal OR Somalia OR South Africa OR Sudan OR Swaziland OR Tanzania OR Togo OR Uganda OR Zambia OR Zimbabwe) |
| CINAHL (EBSCOhost) | 250 | (Leadership OR Governance OR Supervision OR Coordination OR Healthcare Management) AND (MNCH OR Maternal Health OR Child Health OR Newborn Health OR Neonatal Health OR “Maternal Child Health Services”) AND (Community-based health OR PHC OR “Primary Health Care”) AND (Sub-Saharan Africa OR SSA OR Angola OR Benin OR Botswana OR Burkina Faso OR Burundi OR Cameroon OR Cape Verde OR Central African Republic OR Chad OR Comoros OR Brazzaville OR Congo OR Côte d'Ivoire OR Djibouti OR Equatorial Guinea OR Eritrea OR Ethiopia OR Gabon OR Gambia OR Ghana OR Guinea OR Guinea Bissau OR Kenya OR Lesotho OR Liberia OR Madagascar OR Malawi OR Mali OR Mauritania OR Mauritius OR Mozambique OR Namibia OR Niger OR Nigeria OR Rwanda OR “Sao Tome and Principe” OR Senegal OR Somalia OR South Africa OR Sudan OR Swaziland OR Tanzania OR Togo OR Uganda OR Zambia OR Zimbabwe) |
| Embase | 13 | (Leadership OR Governance OR Supervision OR Coordination OR Healthcare Management) AND (MNCH OR Maternal Health OR Child Health OR Newborn Health OR Neonatal Health OR “Maternal Child Health Services”) AND (Community-based health OR “Primary Health Care”) AND (Sub-Saharan Africa OR SSA OR Angola OR Benin OR Botswana OR Burkina Faso OR Burundi OR Cameroon OR Cape Verde OR Central African Republic OR Chad OR Comoros OR Brazzaville OR Congo OR Côte d'Ivoire OR Djibouti OR Equatorial Guinea OR Eritrea OR Ethiopia OR Gabon OR Gambia OR Ghana OR Guinea OR Guinea Bissau OR Kenya OR Lesotho OR Liberia OR Madagascar OR Malawi OR Mali OR Mauritania OR Mauritius OR Mozambique OR Namibia OR Niger OR Nigeria OR Rwanda OR “Sao Tome and Principe” OR Senegal OR Somalia OR South Africa OR Sudan OR Swaziland OR Tanzania OR Togo OR Uganda OR Zambia OR Zimbabwe) |
| Scopus | 73 | ALL (leadership OR governance OR supervision OR coordination OR healthcare AND management) AND ALL (mnch OR maternal AND health OR child AND health OR newborn AND health OR neonatal AND health OR "Maternal Child Health Services") AND ALL (community-based AND health OR "Primary Health Care") AND ALL (sub-saharan AND africa OR ssa OR angola OR benin OR botswana OR burkina AND faso OR burundi OR cameroon OR cape AND verde OR central AND african AND republic OR chad OR comoros OR brazzaville OR congo AND cote AND d´ivoire OR djibouti OR equatorial AND guinea OR eritrea OR ethiopia OR gabon OR gambia OR ghana OR guinea OR guinea AND bissau OR kenya OR lesotho OR liberia OR madagascar OR malawi OR mali OR mauritania OR mauritius OR mozambique OR namibia OR niger OR nigeria OR rwanda OR "Sao Tome and Principe" OR senegal OR somalia OR south AND africa OR sudan OR swaziland OR tanzania OR togo OR uganda OR zambia OR zimbabwe) |
